# “I miss the normalness”: Mother and child perspectives of well-being and effective remote support from primary schools during Covid-19 school closures

**DOI:** 10.1186/s40359-023-01260-w

**Published:** 2023-08-03

**Authors:** Alison J. Lacey, Robin Banerjee, Lucie Dockalova, Kathryn J. Lester

**Affiliations:** School of Psychology, University of Sussex, Falmer, Brighton, UK

**Keywords:** Covid-19, Well-being, Whole-school approaches, Primary education, Social development

## Abstract

**Background:**

Covid-19 related school closures radically disrupted children’s access to social and educational opportunities and changed daily life for millions of families across the world. Emerging evidence indicates that, overall, closures were associated with a decline in children’s mental health and well-being, although individual experiences varied widely. The extent to which primary schools adapted remote well-being support is likely to have contributed to child and family adjustment, although this has not yet been fully explored in Covid related research.

**Methods:**

This longitudinal qualitative study examines variability in remote well-being provision in primary schools during the pandemic, and following school reopening, from the perspective of mothers and children using a whole school approaches framework. Twenty-one primary school aged children and their mothers took part in a semi-structured interview at two time points: time one during the first UK national lockdown and time two approximately seven months later after most children had returned to school. A hybrid inductive-deductive thematic analysis approach identified key themes relating to trajectories of well-being and remote school approaches over this period.

**Results:**

School closures were associated with a decline in well-being for most children. Disrupted contact with friends and teachers, and limited opportunities for enriched, meaningful activity were identified as key risk factors. Protective factors included family and child characteristics that mitigated against the loss of wider systems of support, including family socioeconomic status, parental availability, child temperament, and structured daily routines. Four key dimensions of effective remote well-being provision were identified (the 4Cs). The 4Cs - contact, content, creativity and community – provide an accessible framework for schools to foster children’s social relationships and sense of belonging during periods of closure. Analysis of pupil reintegration outcomes suggest that post-Covid support priorities should include extending social and play-based universal interventions in schools.

**Conclusion:**

Remote well-being support for children during Covid-related school closures varied in quality with implications for children’s mental health and well-being. Findings from this study highlight the importance of ongoing social contact and enriched daily activities to protect children’s well-being and development and present a framework of effective remote wellbeing support for primary schools in the event of future closures or prolonged pupil absence.

**Supplementary Information:**

The online version contains supplementary material available at 10.1186/s40359-023-01260-w.

## Introduction

In March 2020, in an attempt to reduce the spread of the Covid-19 pandemic, the UK Government announced the closure of schools to all pupils apart from those identified as vulnerable and the children of key workers as part of its national lockdown strategy. Similar disease mitigation strategies were used throughout the world with an estimated 107 countries implementing closures impacting over half the global student population [1].

Emerging evidence demonstrates that school closures have had a negative impact on children’s educational progress with global learning loss estimates of between 0.3 and 0.9 years [2]. Moreover, this learning loss is likely to have been particularly acute for children from disadvantaged backgrounds or those who already struggled at school. A rapid review by the UK Education Endowment Fund suggests that school closures may have widened the attainment gap between disadvantaged children and their peers by a median estimate of 36% [3].

Children’s emotional well-being and mental health is also likely to have been negatively impacted by closures although research evidence on this is more mixed. A recent systematic review commissioned by the UK Government’s SAGE group (Scientific Advisory Group for Emergencies) associated school closures with a considerable increase in children’s emotional, behavioural and attention difficulties and general distress [1], while another reported increased rates of depression and anxiety, especially for girls and children with identified additional risk factors [4]. The cumulative effects of social isolation and loneliness have also been associated with elevated risk of depression and anxiety in children [5, 6]. However, other studies indicate that the impact of lockdown on children’s well-being has been more nuanced with some reported benefits. One recent large-scale study reported that while conduct and hyperactivity problems increased over time, internalising emotional problems remained stable and even decreased slightly [7]. Another study reported overall well-being benefits including improved sleep and happiness during the period of national lockdown [8].

These mixed results suggest heterogeneity in children’s experiences during school closures that are likely to be associated with the impact to children’s wider systems. Bronfenbrenner’s Ecological Systems Theory [9] provides a useful framework to consider the disruption to children’s social systems during this time. In normal circumstances, primary school aged children access multiple, intersecting developmental systems, including schools, friendship networks, and the wider community. The extent to which families were supported to mitigate the negative effects of radical system disruption during the pandemic is likely to have influenced children’s outcomes over this period. Whether an individual child thrived or suffered, for example, is likely to be determined by multiple contributing factors, including (but not limited to) family environment, social networks, access to enriched leisure experiences [10], and the extent to which connections were maintained with wider systems of support, including schools. We know that high quality well-being support in primary schools has a positive impact on children, in particular in helping to foster feelings of connectedness and belonging [11]. However, to date very little research has focused on the ways in which primary schools adapted the provision of well-being support during the period of prolonged closures and as schools began to reopen to pupils, and the impact of this on children’s well-being and family functioning [12].

Well-being support in primary schools is increasingly delivered using a whole-school approaches model (WSA). In the UK, WSA is recommended by the Department for Education [13] and forms part of statutory teacher training. It is also in common use throughout the EU [14] and the USA [15]. A whole-school approach describes a governing ethos that recognises the interconnectedness between academic attainment and pupils’ health and well-being [16], and which views the promotion of pupils’ emotional and social well-being as a core school responsibility [17]. Whole-school approaches recognise that schools are more than sites of learning and seek to promote children’s social and emotional well-being using a combination of targeted and universal interventions embedded within a connected and responsive school system [18]. This includes an emphasis on developing positive relationships with families and an explicit role to develop identified protective factors and minimise risk factors, including disadvantage at home [19].

Whole-school approaches offer a useful framework to evaluate the role and remit of primary schools during Covid-19 and to consider the extent to which these wider responsibilities were integrated into remote provisions for pupils. The model also provides a useful opportunity to go beyond a post-hoc assessment of school performance during the pandemic to consider ways in which whole-school approaches could be usefully adapted and modified to incorporate new learning about how children and families adapted over this period.

Although there is a growing body of research exploring the quality of home learning materials provided by schools during Covid-19 related closures [20], and the associated impact on learning [21, 22], to date there has been relatively little focus on the significance of schools’ well-being response and its impact on pupils at home during closures, despite studies indicating that this is a primary concern for teachers [23]. Exploratory qualitative research is essential to understand the nuanced ways in which children and families were impacted by the sudden and drastic changes caused by school closures and to identify how schools most usefully supported children and families during this period. Longitudinal qualitative research in the context of the Covid-19 pandemic, in particular, allows us to explore how change was differentially experienced by individuals in response to moments of upheaval and transition [24] and to build up a picture of patterns of growth and resilience that will usefully inform future development of school-based well-being interventions.

### The current study

Using a longitudinal design, this study qualitatively explores the impact of school closures on children’s mental health and well-being as well as variability in approaches to well-being support both during school closures and following reopening from the perspective of parents and primary school aged children. We broadly define well-being support as non-educational input to foster a positive learning environment for children and to promote social and emotional well-being and resilience. This dual perspective design offers important insights into children’s experiences during a time when oversight of children and families was low [25]. The inclusion of children’s voices addresses an important research gap: despite being disproportionately impacted by Covid-19 restrictions [26], very little research to date includes children’s perspectives and experiences [10].

We interviewed mothers and primary school children from three adjacent local authorities in the UK at two time points. This approach allowed us to explore variability between schools that were subject to similar local authority restrictions and guidance. It also enabled us to develop place-based recommendations for schools in these areas based on our findings (see appendix A). To ensure children had sufficient cognitive maturity to take part, we recruited pupils who were in years 3–6 (7–11 years old) during the study (academic year 2020/21). Semi-structured interviews were designed to address broad research questions around the impact of school closures on children’s mental health and well-being, and any associations with variability in well-being support between schools. In the second phase we explored the effectiveness of schools’ response to reintegration after national school reopening in the context of children’s social and emotional well-being outcomes.

## Method

### Participants

The study was open to parents/guardians of children enrolled in years 3–6 (7–11 years old) during the academic year 20/21. Eligibility criteria required children to be (i) enrolled in a state (publicly funded) primary school in East Sussex, West Sussex, or Brighton & Hove; (ii) not eligible to attend school due to keyworker or vulnerable child status; and (iii) considered typically developing. Of the 21 mothers who took part, 16 (76%) were White British; three (14%) indicated other white backgrounds; and two (9%) participants were Asian/Asian British. This represents a slightly greater ethnic diversity than local and national averages (local population 85% White British compared with 79% nationally) [27]. Eighteen mothers (86%) were either married or co-habiting. Overall, the sample represented a range of socio-economic backgrounds with 12 mothers (57%) reporting household income above the UK national median (£30,800) [28] and 18 (86%) having obtained an undergraduate degree or higher.

Of the 21 children who took part, 18 (86%) were White British and three (14%) were described as having mixed/multiple ethnic backgrounds. Ten (48%) children were female. Child age ranged from 7 years 0 months to 10 years 8 months (*M* = 9 years 0 months, *sd* = 1 year, 2 months). During the academic year 2020/21, four children (19%) were in year 3 ( 7–8 years old); five (24%) were in year 4 (8–9 years old ); six (29%) were in year 5 (9–10 years old); and six (29%) were in year 6 (10–11 years old).

### Procedure

#### Time 1

Parents/guardians were invited to register interest for the study via advertisements placed in local on-line parenting forums in three local authorities in southern England during July 2020. Recruitment posters included a digital link to a study information sheet and consent form, and parents were informed that they would be contacted within two weeks about whether they had been selected to take part. In total, 43 parents registered to take part between 7 and 11 July 2020, all of whom were mothers. Of these, 21 dyads were selected for interview using stratified random sampling to ensure equal representation of girls and boys, and all eligible school year groups. Selected participants were contacted by phone to schedule the interview via MS Teams and to go through the information sheet and consent form, with opportunities for participants to ask questions. Parents who were not selected for interview were contacted via email to thank them for their interest. All interviews took place during July 2020; four months after schools closed to most pupils in the UK. Interviews were conducted by one of two researchers [AL and LD] using a semi-structured interview guide. To ensure consistency, both interviewers met to discuss interview approach before data collection, and LD observed a pilot interview with a volunteer parent/child dyad that was led by AL.

Child interviews were approximately 15–20 min long, and parents were invited to stay to support their child if desired. In total, 16 mothers opted to be present and five left the room. Children were asked to reflect on their experiences at home during lockdown, including impact on friendships, and to describe any support they had received from school. They were also asked about their feelings and expectations about going back to school in September 2020. Mothers were asked to consider the impact of school closures on their child’s social and emotional well-being and to evaluate their school’s response to supporting families at home. Finally, mothers were asked to describe their feelings about schools reopening in September 2020 and to highlight any issues they felt should be prioritised to support successful reintegration. Parent interviews were approximately 45 min long.

#### Time 2

All 21 families consented to take part in a second interview during December 2020 and January 2021, at which point all schools in the UK had been reopen since September 2020. Interviews took place between 14 December 2020 and 6 February 2021. During this period the UK Government announced a second period of national school closures. Interview topic guides were adapted to include additional questions about the impact of this change on children and parents. The 10 families who had completed the interview prior to the Government announcement were invited to provide responses to the additional questions in writing via email. Of these families, seven provided email responses.

All dyads were interviewed by the same interviewer as at Time 1. Semi-structured interview guides were developed to elicit responses about the ways in which schools had supported pupil reintegration during the Autumn term. Questions were based on an established whole-school approaches conceptual framework identifying four levels at which schools structure support for children’s social and emotional well-being: individual pupil, small groups, class, and whole school [16]. Mothers and children were asked to reflect on how children had adjusted to going back to school, including describing any significant changes. Participants were also asked to describe ways in which class teachers and the wider school community had helped children to settle in at each of the four levels in the conceptual WSA model [16]. Dyads interviewed after the announcement of the second period of closures were asked to describe their thoughts and feelings about this, including any changes to the support and home learning materials offered by schools.

Interviews were digitally recorded and transcribed verbatim. All identifying information was removed during transcription and each child was given an alias. Aliases are used to identify individuals throughout this report, followed by Y3-6 to indicate school year group.

### Data analysis

#### Time 1

Transcribed interviews were analysed using reflexive inductive thematic analysis using a critical realist approach [29]. To ensure that the perspectives and experiences of each dyad were accorded equal priority, each transcript was analysed inductively using non-hierarchical initial codes. Patterns were then synthesised across the dataset in a recursive process that included frequent checking back to the original data to ensure that identified themes provided an accurate and meaningful account of the data [30].

Inductive thematic analysis is an active interpretative process and, as such, it is important to recognise the position of the researcher in the context of data analysis. Two authors (AL and KL) are parents of primary school aged children who were themselves directly impacted by school closures. While this personal experience supported the building of rapport, we were mindful of the potential of these experiences to impact data collection and analysis. To minimise the risk of researcher bias, throughout analysis the research team met fortnightly to discuss our own assumptions and experiences and to interrogate analysis in the context of those experiences. Final themes were reviewed and agreed jointly by the main research team (AL, RB and KL).

#### Time 2

To allow a nuanced consideration of change over time we employed a hybrid approach to analysis at time 2 incorporating both inductive and deductive thematic coding [24, 31, 32]. The thematic structure from Time 1 was imported as a flexible deductive framework from which to analyse transcripts at Time 2. New codes were incorporated where appropriate to represent inductively coded new or emerging themes. This approach ensured our interpretative analysis had a central focus on change over time (within person narratives) as well as on between subject comparisons at each time point [31].

Finally, identified themes across timepoints were organised into four super-ordinate latent themes. Throughout analysis the research team met fortnightly to consider, and reach agreement on, identified themes.

## Results

Table 1 sets out the themes and sub-themes identified, along with a brief description.

**Table 1:** Summary of themes

Theme	Description
**1: Negative impact of school closures on pupil well-being** 1.1 Mood and behavioural decline (Time 1) 1.2 Improved child mental health and well-being following school reopening (Time 2)	Describes child well-being trajectories during school closures and following school reopening in September 2020.
**2: Factors influencing children’s well-being** 2.1. Family availability and resources 2.2. Social contact with peers 2.3. Activity and routine 2.4. Resilient and adaptable children	Identifies key protective factors for child well-being during periods of school closures, including child and family characteristics
**3: The 4Cs of effective remote well-being support** 3.1. Contact 3.2 Content 3.3. Creativity 3.4. Community	Identifies four key areas for effective remote well-being provision during periods of school closures
**4: Reintegration: reflections and priorities** 4.1 Targeted support 4.2 Universal support 4.3 Significance of children’s wider systems	Parent and child reflections on the role of schools in promoting child well-being and development.

### Theme 1: Negative impact of school closures on pupil well-being

#### Mood and behavioural decline (Time 1)

Most children experienced mood and behavioural decline during the first phase of closures. For many this decline was described as significant and prolonged with lockdown associated with feelings of listlessness and ennui, as well as anger and frustration. One child described lockdown as “empty time” [when] “I sit on my bed and I get angry with my sisters” (Seb, Y4). Feelings of loneliness and isolation were particularly acute for children whose parents and siblings were unavailable for long periods due to work and school commitments: “At school you get to talk to your friends…but at home my parents are working, my sister is doing her own thing, so I’m kind of like on my own” (Evie, Y6).

Lack of contact with friends and teachers was identified as a leading cause of distress by all children we interviewed, along with missing normal school routines and activities: “I miss the normalness and I miss the fun of being with my friends and my teacher” (Peter, Y4). Mothers also attributed children’s mood decline to disrupted social networks and routines, as well as describing increased pressure on family relationships caused by being “intensely together” (Mabel’s mum, Y3) in an environment of elevated stress: “Families have been through a really difficult time…at times our home has been like a pressure cooker” (Frank’s mum, Y6).

For some children, mood decline was so significant that parents negotiated an early return to school under vulnerable and/or keyworker provision. “It just went downhill quite quickly… It was quite frightening and if she hadn’t been able to go back to school, I don’t know what we would have done.” (Evie’s mum, Y6). “My kid was depressed! He was ten years old with symptoms of a quite serious depression. It was heart breaking. I had to email the school and say I really needed him to come in” (Frank’s mum, Y6).

Children with less severe symptoms were described as more “up and down” (Peter’s mum, Y4) than usual with increased frequency of tantrums and emotional meltdowns, “lots more extreme crying – seemingly about nothing” (Nicole’s mum Y4) or who developed new anxiety symptoms that parents associated with not being able to take part in normal social activities: “He’s developed this swallowing tic that he didn’t have before…because…you know, he has big ideas and feels like we’re pouring cold water on them all the time” (Seb’s mum, Y4).

#### Improved child mental health and well-being following school reopening (Time 2)

Of the children who experienced mood and behavioural decline at Time 1, most experienced a full recovery following the reopening of schools, including the children whose symptoms were severe enough to warrant an early return to school. Children described feeling joy at being with their friends again and being back in a stimulating, familiar routine: “The first day back I was thinking, ‘This is back to school! This is back to school, and it’s great because I don’t have to be in that annoying old lockdown’” (Seb, Y4). For some parents, the change in mood was so marked it felt like having a different child in the house: “It’s like somebody came and lived here for lockdown and now I have my Ada back” (Ada’s mum, Y5).

Like children, mothers attributed mood and behavioural improvements to reconnecting with friends and being back in a structured, stimulating learning environment: “It’s so much better. He needs to be out. He should be with his friends. He’s not designed to be locked up!” (Toby’s mum. Y3).

Partial well-being improvements were reported for the remaining children who experienced mood and behavioural decline at Time 1. While these children enjoyed many aspects of returning to school, some residual anxiety or behaviour issues remained with some children finding it more of a challenge to re-establish positive friendships, particularly within the context of ongoing Covid restrictions in school: “…it started to dawn on her that it was far from normal. The novelty and the excitement of it wore off, combined with the exhaustion of having to readjust to the structure” (Mabel’s mum, Y3).

Overall, we found that school closures were associated with mood and behavioural decline which, for most children, resolved without the need for additional intervention following school reopening. The spontaneous nature of this recovery supports the attributions of both parents and children that a decline in well-being over this period was in large part related to the disruption to important social systems, including social relationships and access to enriched activities and routines.

### Theme 2: Factors influencing children’s well-being

Although lockdown was associated with mood and behavioural decline for most children, several protective factors were identified that predicted well-being outcomes over this period.

#### Family availability and resources

Parental availability, confidence, and access to financial resources were important indicators of well-being, with families who were able to spend time supporting children with home learning, or those able to provide enriched lockdown activities, less likely to report a marked deterioration.

We’ve got big house and she’s got two parents and we haven’t struggled for money. Um, you know, all this kind of stuff. And I think there’s going to be significant numbers of children—her school isn’t particularly well-off—who’ve had a shocking time (Nicole, Y4).

The requirement for parents to assume the role of teacher reduced time available for parents and children to spend time together having fun and was a significant “source of tension” (Ben’s mum, Y3) for many families, with both parents and children associating the adjustment to normal parent-child relationships with increased stress and conflict. Working parents and those lacking teaching confidence or skills found this change particularly challenging.

I’m not a teacher, I can’t—I can’t—I’ve looked at some of it and it’s the way they learn things as well. I’m trying to explain something to her and then she’s like—that’s not how we learn it. And I’m like—well, I don’t know how you learn it at school (Ellie, Y5).

Parental availability due to the pressures of working from home was another important factor influencing well-being with both mothers and children describing parents being physically present but emotionally unavailable due to work commitments as particularly emotionally demanding: “I wish that mum and dad would be more with us because they’re usually working” (Peter, Y4).

School closures and lockdown shifted the way families occupied the home with a blurring of boundaries between the domestic sphere and parents’ professional and public roles. Mothers recognised that this shift was often difficult for children whose homes were no longer private spaces but workplaces and schools, and who were often cut off from contact with parents for prolonged periods during the working day: “I might have two or three meetings where they can’t come to the door at all—they’ve found that quite tough, actually, because this is their family home, this is their kitchen, you know” (Lily, Y4). “It was really hard because Daddy was here but not with him…It was easier when Daddy was in the office, than—than him being at home but not being involved” (Toby’s mum, Y3).

In the wider context of reduced play and social opportunities, sibling relationships became increasingly important. Mothers described the play and companionship afforded by sibling relationships as important protective factors and that overall siblings were more bonded and had “been able to enjoy each other more” (Lizzy’s mum, Y3). Conversely, children without siblings were more likely to experience pronounced feelings of loneliness and isolation which put additional pressure on parent and child relationships: “He’s an only child as well—we try to not make him too isolated…It’s been hard being alone and I think he missed a lot the– well the social aspect” (Toby’s mum, Y3).

Finally, children with a family member particularly vulnerable to Covid-19 were more likely to experience feelings of anxiety, especially during the phased reopening of public facilities: “because I haven’t been able to sort of take her out to the parks and go and see her friends and um, that’s kind of made it like an extra level of difficult” (Florence’s mum, Y6).

#### Social contact with peers

Ongoing meaningful contact with peers, as well as with extended family and friends, were important predictors of emotional well-being. Children described missing spontaneous, physical play with friends in the playground at school or in the park as a big loss: “[The worst part of lockdown is] missing my friends and then every play—running out into the playground to get there first to play football” (Toby, Y3). Parents noticed improvements in children’s well-being when public play areas reopened and children were permitted to play outdoors: “because lockdown has eased a bit, we’ve now been able to meet up with friends more regularly and go out which has been really good for him” (Theo’s mum, Y5).

All 21 children and mothers cited lack of in-person contact with friends as the primary reason for feelings of loneliness, frustration and lack of motivation, with mothers tending to report more concern about children’s social relationships than educational progress: “I’m not bothered about the schooling aspect. He’s seven. But the, um, the friend aspect is…” (Toby’s mum, Y3).

Mothers were also concerned about the longer-term impact of social isolation on children’s social competence and emotional well-being, with several noticing a decline in social skills which they attributed to disrupted peer contact: “It was very obvious, like the first time he met up with people that they were kind of like a bit ‘Ahh! You know, what do we do?” (Theo’s mum, Y5).

Opportunities to meet up with friends took on a new significance during lockdown, with parents describing an enhanced appreciation of the value of play for children’s development.

Play dates for me have always been ‘oh yes, I ought to arrange them’ because it’s really difficult with work. And now I’m like, oh no this is really priority, so I’ve been texting a friend…I’ve said, ‘please can we come over this week, let me know I’ll bring him’. And I cried when they said they were up for it because he needs it so much [getting tearful] (Seb’s mum, Y4).

While online platforms like Zoom or FaceTime were commonly used by parents to try and maintain friendships, these tended to be less meaningful for younger children or for children whose friendships are primarily based on physical play. Children tended to agree that while virtual contact was better than nothing it was not a good substitute for in-person interactions: “I just felt it was nice to see her again…I didn’t really get to play with her, but it was better than not seeing her at all” (Wes, Y4).

The most successful remote social interactions were between children with good conversational abilities and who had a strong existing friendship. Shy children, and those with low social competence, were less likely to be able to successfully manage remote interactions with friends. Online contact was also less successful for new or emerging friendships, or between children who found extended conversations difficult or boring.

It was kind of a little bit tantalising. At their age, they don’t really want to be like making conversation, they want to be playing football or like running around. So, I think sometimes he came away from those Zoom conversations feeling like a little bit empty and a little bit worse (Frank’s mum, Y6).

Group interactions were also very challenging online, with most families reporting abandoning attempts at whole class, or large group meetups, because they were too difficult for children to manage. Parents felt that initiating online interactions required a level of social confidence that often excluded reticent or younger children.

It was very difficult to have a group conversation where everybody to talks and, um, yeah, he doesn’t necessarily like all the attention on him. So, to speak in front of everyone when it was just one-to-one but with the whole group listening, he kind of didn’t really want to say an awful lot (Theo’s mum, Y5).

#### Activity and routine

Maintaining a predictable and varied daily routine was important for children’s well-being, with parents commenting that being outside in nature, engaging in sport and exercise, and having something meaningful to do were essential for maintaining motivation and a positive outlook: “It’s worse, I think, emotionally, if you just let them do nothing all day” (Lily’s mum, Y4). Families with the time and resources to go out for regular walks, or who were able to plan a range of enhanced activities were more likely to report benefits of lockdown for children: “Even when the lockdown was really strict, we could still walk somewhere and, you know, be out for half the day and not—not come in contact with many people” (Theo’s mum, Y5). Parents felt that being outdoors had therapeutic value for children experiencing low mood or poor motivation and described prioritising getting children outside despite work commitments because of the impact on mood and behaviour. “It was just reminding him that there was an outside world…we were looking at the colours of the rainbow and trying to find flowers every colour of the rainbow, um he really picked up” (Daniel’s mum, Y5).

#### Resilient and adaptable children

Children’s temperament, resilience and social competence were identified as important factors influencing children’s well-being during closures. Children described as emotionally articulate were more likely to regulate their feelings and emotions and communicate their well-being needs to parents: “…she’s quite mature and she’s quite articulate so she’s been able to express how she’s been feeling” (Lily’s mum, Y4). In contrast, children with less developed emotional intelligence had more difficulty understanding their own feelings, and positively framing their experiences: “It’s quite hard for him to process it or articulate it because I think when you ask him those questions, he doesn’t necessarily even know how it has affected him but observing him…I would say that it really, really has” (Frank’s mum, Y6).

Children who coped best with lockdown were described as self-motivated and able to make the most of the sudden increase in unstructured free time. The ability to respond creatively to boredom and the enforced slowing down were also indicators of positive adjustment: “He’s been pottering around finding things to do on his own and actually he’s not found it very difficult” (Ben’s mum, Y3).

We got the mouldy bug-eaten sun loungers out of the shed but dusted all the bugs off them and now we—every time it’s sunny, we get them out and lay on them. It’s nice to watch the clouds float by (Peter, Y4).

Overall, risk and protective factors tended to mirror existing indicators of social disadvantage. Children who thrived were more likely to have families with access to financial and practical resources to scaffold lockdown activities to help children adjust to their changed environment. The only family-level risk factor that did not fit this pattern of existing disadvantage was parent availability, highlighting the significant additional pressures experienced by working parents during this time, and the complex challenge of balancing work, childcare, and school responsibilities for parents working from home. Maintaining relationships with friends, and connections with teachers was also important, with children’s temperament, age, and social competence influencing the extent that this was possible within the limitations imposed on social interactions.

### Theme 3: The 4Cs of effective remote well-being support

There was significant variability in the ways in which schools maintained focus on children’s social and emotional well-being during closures, with parents describing a general loss of clarity about well-being support. This theme describes the identification of the 4Cs: four key features of effective remote support identified by both mothers and children as supporting children’s well-being and engagement at home. Appendix A includes a series of infographics based on the 4Cs which were distributed to local schools during the second period of closures.

#### Contact

Irregular or inadequate contact with families at home was a leading concern with parents feeling that lack of contact with a known teacher prevented meaningful assessment and monitoring of child well-being. Overall, parents felt that schools had been slow to respond to the needs of children at home at the cost of essential safe-guarding responsibilities: “After a month of being in lockdown they don’t know who their vulnerable children are any more” (Ada’s mum, Y5).

There will categorically be kids out there who have been absolutely neglected for months and months and they will have been in a terrible situation with their families going nuts and I don’t think the school would necessarily have picked up on that (Frank’s mum, Y6).

Where regular contact was in place, parents did not feel this was always delivered in the most meaningful or appropriate way, with mothers frustrated by generic communication strategies or a one-size fits all approach. Direct contact between children and a known teacher was felt to be the most valuable strategy overall.

They were sending out these well-being and inclusion newsletters and I was just thinking these are a complete waste of time… I just didn’t think they really addressed that very well. The thing that everyone wanted was that personal contact, even just... they could have done a 20-minute phone call once a week or you know, that’s totally do-able (Laurie’s mum, Y6).

Families who described having predictable and regular contact with teachers felt that it had contributed significantly to children’s motivation and engagement, particularly if contact included the facilitation of peer interactions: “She’s had a weekly Zoom with her class. It felt like she was still part of something” (Nicole’s mum, Y4). “He really perked up after [speaking to the teacher]…and her giving some motivational…praise…I would see him smiling and he was full of beans after that” (Daniel’s mum, Y5).

#### Content

The quality of home learning provision also varied widely. Parents were most satisfied with schools that provided manageable and accessible resources with regular teacher feedback and some opportunity for peer learning via live online teaching, commenting that this connection made activities feel less monotonous and isolating: “they’re logging on, you can actually see their faces which has been really great and it’s like, nice to see more groups and Sophie can then see her classmates and catch up with her teacher which is—she’s really loved” (Sophie’s mum, Y5). Although most parents recognised the challenge of providing a rich and varied remote curriculum, many were critical of a perceived failure to adequately account for children’s need for dynamic feedback, variety, and the opportunity to learn with peers. Parents associated tedious or repetitive home learning materials, or a very restricted focus on core skills, with a decline in children’s well-being. Overall, most families were dissatisfied or ambivalent about the content of home learning and were critical of a perceived reluctance for schools to provide interactive teaching online: “Other organisations have adapted but the schools just couldn’t seem to get their heads round how to do it” (Laurie’s mum, Y6).

What struck me was that we’re expecting her to work as an adult with even less support and motivation than an adult would get. She’s not getting an appraisal…she’s just having to sit in front of a computer doing almost a data entry job for hours at a time (Evie’s mum, Y6)

Children also tended to talk about home learning in negative terms, describing tasks as a poor substitute for normal lessons and reported missing regular input from teachers and opportunities to share their learning with peers: “There’s not much I’ve been enjoying about not going to school to be honest. I haven’t really been enjoying home schooling” (Harry, Y5).

During the second period of school closures, parents and children reported significant improvements to home learning resources which they related to increased child engagement. Mothers felt that learning plans had a more coherent and manageable structure, and most children were taught via pre-recorded videos or live on-line lessons rather than worksheets.

My home learning was much harder the first time we were off because we didn’t know what we were doing, and it was all confusing. But now this lockdown it’s much easier (Florence, Y6).

However, despite improvements, parents were concerned that pupils’ well-being was likely to be adversely impacted by a second period of home learning with virtual learning considered no substitute for face-to-face interactions.

It feels like care-taking a little bit. I think it will be incredibly challenging for them to really see where children need specific help…You know, probably kind of giving them lots of food and hoping that they eat it rather than actually having a conversation while they eat it and teaching them how to eat better, you know (Seb’s mum, Y4).

#### Creativity

Some schools came up with imaginative ways to engage children during closures, and parents particularly valued varied activities that children could access independently and that kept them imaginatively occupied.

There was one week …superhero week and it was absolutely brilliant. They sent home so many fun things to do. They had music, they had art and design, they had like—make a superhero costume, write a story, do a comic strip. There was just—and my kids wanted to do all of it. That was a really fun week. We just did the whole lot, and everybody was posting, and it was really cool, we could see what was going on… (Frank, Y6).

However, overall, parents felt that creative opportunities to inspire and motivate children beyond the curriculum had been missed and that there should have been a stronger emphasis on “the social aspects of learning and fun” (Nicole’s mum, Y4). Several parents attributed this to a lack of ambition from schools and policy makers over this period. “The starting point is always “we can’t rather than, can we?” (Mabel’s mum, Y3). “The kids… at home, they’ve been pretty much abandoned. Or that’s what we feel like” (Frank’s mum, Y6). Overall parents felt that support for children at home had not been schools’ main priority. Specifically, they identified a lack of coordinated planning and resources that could have enabled schools to provide a more ambitious programme of support with a holistic focus on children’s well-being. “I do think the whole thing was inevitable, given the complete lack of preparedness of the Government to deal with any of this” (Evie’s mum, Y6).

#### Community

Schools also varied in the extent they were able to maintain a sense of community and connectedness with families. Most parents felt that schools had a duty to support and promote emotional and social well-being and were critical of schools perceived as adopting a restricted academic focus. Overall, parents felt that children living in disadvantaged environments would be disproportionately impacted by a’ perceived reluctance to maintain a connected school system for families at home: “[There should be] recognition of the fact that schools should provide more than education” (Evie’s mum, Y6). “It’s not just about the work but the connection. Just reminding people that they care about you. That’s important” (Harry’s mum, Y5).

Examples of good practice included hosting virtual, or distanced, community events and competitions, providing remote well-being sessions in small groups, and sending supportive messages or creative tasks home. Parents felt that it was important for children to feel that teachers were still there for them and that the perception of being kept in mind was an essential part of maintaining motivation and engagement. “She felt like her teachers were listening, even if they weren’t actually teaching in that time… They showed throughout the whole thing that they cared” (Lily’s mum, Y4).

### Theme 4: Reintegration: reflections and priorities

This theme explores how schools adapted social and emotional learning strategies at each of the four levels identified in the whole-school approaches conceptual framework [16]: individual, small groups, class, and whole school. However, while some schools explicitly prioritised pupils’ social and emotional well-being following school reopening, several parents were unclear about the extent to which well-being was part of the schools’ remit. “I don’t know how much the school feels that they’re really responsible for the emotional well-being of the children. It’s not clear to me that they see that as part of their role” (Frank’s mum, Y6).

#### Targeted support

Most parents were not aware of any targeted interventions specifically to address pupil’s social and emotional well-being although many felt that additional support was likely to be available for pupils on a targeted basis if needed. Where pupils did receive specific interventions, these were either 1:1 emotional therapeutic support from a counsellor or, more frequently, class-based activities talking about their feelings about school closures and lockdown. In general, children did not enjoy explicitly Covid-focused well-being lessons: “We had like one or two class meetings where we talked about like how Covid has been for you and whether you think Covid has affected your family and stuff. But we all found that really boring” (Seb, Y4).

While parents agreed that targeted interventions were necessary for some children, they shared children’s concerns that specific Covid-focused interventions at the class level might not always be helpful, noting that discussing feelings about lockdown was an adult-centric approach, and that children would be less likely to be able to meaningfully access this kind of support. “I think they did bubble times, that’s what they call it in their classes…He’s a bit like, ‘oh you’re here to like draw our feelings again” [laughter] (Toby’s mum, Y3)

#### Universal support

Overall, there was consensus that children’s reintegration was best supported by universal, preventative approaches that focused on developing the school community, promoted children’s participation in outdoor learning and sport, and prioritised fun and play.

Access to play was identified as an important way to support children’s friendships and well-being. While parents generally felt that schools had managed to balance ongoing Covid restrictions with children’s access to play, several were critical of perceived unreasonable restrictions to play time and equipment which they felt came at a cost of allowing children to settle back into school routines and social groups.

I think the restrictions put on how they were allowed to play and the space they were allowed to use. You know, I think it’s difficult, isn’t it? Having, like, your free time constrained when you’re working really hard? (Theo’s mum, Y5).

This concern was echoed by all the children we interviewed who described frustrations about restricted access to play as the most important limiting factor during the first term back to school. While some schools adapted cleaning schedules to ensure most play equipment remained accessible to children, others had imposed tight restrictions on the use of play areas: “We’re allowed to walk through it [adventure play equipment], but we’re not allowed to actually play on it (Wes, Y4).

Although opportunities for the whole school to gather for assemblies were limited by ongoing restrictions, parents reported that several schools operated virtual or outdoor assemblies and retained a focus on reinstating school routines and fun. One mother described the significant impact of an outdoor concert hosted by her child’s school during this time. “Music is blaring out and I kind of had a bit of a — I felt quite emotional about it, you know—that they refuse to kind of diminish the experience of the children” (Peter’s mum, Y4).

#### Significance of children’s wider systems

The marked improvement in children’s well-being following the return to school was largely attributed to the opportunity for children to interact and play with their friends, and to experience a varied and stimulating routine. Mothers commented that children benefitted from being in larger groups and from learning to be more socially independent, particularly as they approached transition to secondary (high) school.

He’s really enjoyed being with his friends again. Like he just loves being with them. I’ve really noticed now he’s in year 6 as well I think it’s so important that he’s all about his friends. So, he’s always talking about his friends—like his attention has completely shifted to his friends rather than us” (Laurie’s mum, Y6).

For most parents, children being back in school was also associated with improved family relationships, “he has had a break from the intense family environment that he was in before. You know we can’t really escape from each other so that’s been good for all of us” (Harry’s mum, Y5). While a minority of children required additional targeted support, most adapted well and benefitted from the social contact and enriched environment of the classroom. Parents reflected that lockdown and the period of closures had given them new insight into the role of schools and their wider significance for children’s social development and well-being. “I feel very strongly that they need—that for their well-being, school is the place for them to be. And I felt that that overrides really anything else” (Ben’s mum, Y3).

Overall, parents felt that schools, and society more generally, should prioritise children’s access to social contact and meaningful activity in the aftermath of the pandemic, and were concerned about the long-term impact of restrictions on children’s emotional and social development. Parents described emotional health as an essential prerequisite for academic learning and felt that the impact of restrictions on children had frequently failed to adequately consider children’s developmental needs, commenting that the public health response did not properly account for the likely detriment to children’s well-being when making policy recommendations: “I felt that although we weren’t doing things, it was important that if someone was going to go out and do stuff that it should be children” (Theo’s mum, Y5).

## Discussion

This study provides insight into the lockdown experiences of primary school children and their mothers and the role of schools in promoting children’s well-being. The identification of the 4Cs - contact, content, creativity and community - as key protective factors for child well-being provides a framework for effective well-being support during future periods of prolonged school closures, and usefully informs the development of support priorities in schools in the aftermath of the pandemic. Following data analysis, the 4Cs were adapted into a series of accessible infographics to support local schools during the second period of school closures (Appendix A). Analysis of mother and child dyads over time highlights the vital role of schools in well-being promotion, including the importance of regular contact with teachers and peers and universal ethos-level interventions to well-being. Schools that provided a range of rich, stimulating activities for children, and that facilitated a sense of shared community experience and peer support, were highly valued by families and associated with increased engagement and motivation.

Most children we interviewed experienced significant well-being decline during the period of closures which mothers and children associated with social isolation and lack of a varied, stimulating routine. In most cases these difficulties improved spontaneously following school reopening. This aligns with recent evidence identifying lack of social connectedness and meaningful activity as significant risk factors for children during lockdown [4]. It is also supported by research showing that children’s risk of depression is best predicted by the duration, and not the intensity, of social isolation and loneliness [6]. Identified protective factors indicate that continued access to multiple systems of support is essential for maintaining well-being during periods of school closure. Children from families with the resources, time, and motivation to provide enhanced experiences tended to fare better overall, along with children who continued to have some access to outside play with peers. In terms of future planning, these results suggest that a dual focus on both social contact and meaningful activity should be central to supporting children’s mental health and well-being during periods of prolonged school closure or pupil absence, particularly for vulnerable children and families. The 4Cs provides an accessible framework to help school leaders respond dynamically to any future restrictions or to adapt support for pupils who experience prolonged absence due to illness, for example. The 4Cs also suggest a way for schools and policy makers to ameliorate the negative effects of any future social restrictions by enhancing and extending opportunities for stimulating activities and routines, for example through investment in music, outdoor learning, and sport.

Ecological systems theory [9] also provides a useful theoretical perspective to consider these findings. This theoretical model locates the individual child within four separate but inter-connected systems that interact and nurture development. The four systems include the micro system (immediate family and household), the meso system (friends and informal networks), the exo system (schools and other formal networks) and the macro system (economic and political system). During school closures, developmental opportunities through informal networks and schools were radically disrupted leading to increased pressure on the micro system, the family, to fulfil children’s developmental needs. From this perspective, well-being decline was largely due to impoverished access to developmentally important systems, with families with limited access to social, economic, and cultural resources particularly at risk. Significant improvements in mood and behaviour following school reopening can be understood as the consequence of children regaining access to their informal (friends) and formal (school) networks rather than dependent on school-led interventions as such. Indeed, parents and children themselves identified reconnecting with friends as the most important benefit to returning to school.

Findings suggest that social connectedness and meaningful activity, two factors already recognised within whole-school approaches as essential for child well-being [16], were crucial but largely neglected priorities during lockdown. While most parents continued to facilitate social contact and meaningful daily routines for their children despite restrictions, many felt that a coordinated strategy by schools and policy makers was lacking. In our sample, most families were dissatisfied with both the level of contact from schools and the quality of home learning materials. Where schools did engage with pupils at home, either through 1:1 contact with teachers, facilitation of peer learning, or through community events, parents reported improvements in children’s overall motivation and engagement.

There are, perhaps, two important conclusions to be drawn from the identification of social connectedness and meaningful activity as key predictors of positive well-being for children. The first concerns contingency planning for potential further periods of closure and the development of remote whole-school strategies that recognise the centrality of well-being promotion [33]. Clear expectations about recommended remote targeted and universal interventions, including regular, predictable contact with families and the facilitation of peer interactions should be part of an agreed system of accountability in the event of future closures [34] and the 4Cs provide a useful framework for schools to develop effective remote well-being support strategies for pupils. This support should focus on meaningful, regular contact with families; engaging, interactive and differentiated content of home learning materials; creative methods of engaging with children; and opportunities to maintain a sense of community and connectedness. However, there are also implications here for policy makers responsible for balancing public health concerns with risks to the mental health and well-being of children and families. Where possible, any future restrictions should minimise disruption to social relationships and consider ways to safely support children’s ongoing in-person contact with one or more friends.

The second conclusion relates to how whole-school approaches might be usefully adapted within schools following the Covid-19 pandemic. For most children, well-being improved without the need for targeted interventions on the return to school. While we recognise the value and importance of specific, targeted interventions for pupils who need them, findings from this study suggest that there may be important benefits in schools having a renewed focus on promoting social connectedness and meaningful activity within the wider school system. For example, through the provision of creative and dynamic learning beyond the curriculum and by prioritising high, quality interactive play for all pupils [35].

In the context of limited school resources, we know there is a tendency for schools’ to prioritise reactive, deficit-based interventions based on indicated behaviours over preventative, strength-based approaches (33). This is despite the known benefits of universal approaches for children from disadvantaged backgrounds [36] who we know are likely to have been disproportionately impacted by closures [37]. Results from this study suggest that pupil well-being at home and school is best supported by an explicit focus on social connectedness and meaningful activity through enrichment of the school environment through a coordinated universal response, although this should be sensitively balanced with the needs of pupils who are likely to benefit from more targeted interventions, and whose experiences have not been examined in this study.

Whole-school approaches recognise that there is no ‘magic bullet’ intervention [38] but that effective social and emotional health promotion relies on a combination of targeted and universal interventions, with an appropriate balance between well-being promotion and enhanced support for vulnerable, or at risk children [16]. However, while most schools endorse these principles, well-being support in schools has become increasingly specialised over the past 20 years, partly as a consequence of increased teacher workload [16]. One unintended consequence of this could be a narrowing of focus for staff within the school system and a growing imbalance between universal and targeted approaches which may have contributed to the delayed universal response to well-being support during school closures. This delayed response highlights the need for coordinated, strategic leadership to implement and monitor WSA interventions. We know that the most successful WSA programmes acknowledge the complexity of implementation [39]. It may be that school closures will lead to renewed focus on the strategic and resourcing challenges of WSA to ensure more robust and resilient approaches in the future.

### Strengths and limitations

Inferences about child well-being in relation to whole-schools approaches in this study are supported by the dual-informant, longitudinal design. While the sample of mothers and children included a broad range of family configurations and SES, it is important to recognise that, with the exception of one family, none of the families we interviewed were experiencing significant additional stressors, including parental mental health problems, children with additional needs, or significant family conflict. It is likely that recovery may be more complex for children from such families. To address this limitation, we conducted a series of interviews with class teachers and head teachers working in schools serving diverse demographic profiles of familie [40]). There are also limitations relating to convenience sampling via online advertisements. It is likely that recruited participants differed from the general population in terms of interest in the topic area, and willingness to discuss their lockdown experiences. Additionally, although the study was open to parents/guardians, no fathers registered to take part. Inclusion of fathers may have yielded additional insights into the paternal role during this time, particularly as many fathers were working from home. However, emerging research indicates that mothers were disproportionately impacted by school closures and, in heterosexual couples, mothers were more likely to be the ‘default’ parent responsible for managing children’s social and educational activities [41]. Consequently, the experiences of mothers over this period are particularly relevant when considering risk and protective factors relating to children’s social and emotional well-being. Results from this study should be interpreted alongside findings from related research for a more comprehensive overview of the impact of school closures on children.

## Conclusion

This study contributes to growing evidence that school closures had a detrimental impact on children’s emotional and social well-being due to prolonged restrictions to developmentally important networks and systems. Schools have an important role in promoting and facilitating well-being, and it is essential that they are adequately resourced to provide an appropriate range of targeted and universal interventions to extend children’s access to enriched, social learning experiences in the aftermath of the pandemic. Although most children in our study adjusted well following school reopening, the long-term impact on children’s well-being is unknown [42]. This study provides important insight into ways in which schools could best support pupil well-being in the future and in the event of any further prolonged closures.

### Electronic supplementary material

Below is the link to the electronic supplementary material.

Supplementary Material 1

## Data Availability

The datasets used and/or analysed during the current study are available from the corresponding author on reasonable request.
