# Supplementary material for: “I miss the normalness”: Mother and child perspectives of well-being and effective remote support from primary schools during Covid-19 school closures
Source: BMC Psychol. 2023 Aug 3;11:220. doi: 10.1186/s40359-023-01260-w (PMC10401740; doi:10.1186/s40359-023-01260-w)
Supplement: Supplementary file 1 — Supplementary Material 1 [file 40359_2023_1260_MOESM1_ESM.pdf]

Good practice for supporting primary school children at home during school closures

Researchers from the University of Sussex interviewed 21 children in Years 3–6 and their parents about their experiences of home schooling during the first period of school closures (March – July 2020). Parents and children identified four main issues: Contact, Content, Creativity, and Community.

Contact

Regular one-to-one contact with the class teacher or other known member of staff was really important for children's motivation and engagement.

Lack of contact was raised as an important safeguarding concern by some families.

"After a month of being in lockdown they don't know who their vulnerable children are anymore."
Ada's mum, Year 5

"There should have been something more structured and ongoing."
Evie's mum, Year 6

"We felt really connected."
John's mum, Year 6

Parents particularly valued schools that facilitated regular online group meetings between teachers and peers.

Content

Opportunities for live online learning in small groups, or video lessons using pre-recorded materials were valued by both parents and children.

Parents were most satisfied with schools that provided manageable and accessible resources with regular teacher feedback.

"She's had a weekly Zoom with her class. It felt like she was still part of something."
Nicole's mum, Year 4

"Other organisations have adapted but the schools just couldn't seem to get their heads round how to do it."
Laurie's mum, Year 6

"The work is really boring."
Toby, Year 3

Whole school projects to allow coordinated learning between siblings in different year groups were also highly praised.

Creativity

While many schools came up with imaginative ways to engage families, parents identified lots more creative opportunities to inspire and motivate children beyond the curriculum. Access to high quality outdoor learning and play was particularly valued.

"The PTA did a social distanced relay race with kids stopping two metres from each other. 60 kids doing 10k across the neighbourhood."
Toby's mum, Year 3

"Make it more fun and enjoyable, it has been really nice being outside more."
Peter, Year 4

"More play."
Florence, Year 6

"Prioritise the social aspects of learning and fun."
Nicole's mum, Year 4

"The starting point is always 'we can't' rather than, can we?"
Mabel's mum, Year 3

Community

Most parents felt that schools have a responsibility to support and promote emotional, social, and physical well-being and were critical of schools perceived as adopting a restricted academic focus.

"I miss the normalness and the fun of being with my friends and my teacher."
Peter, Year 4

A number of children described feelings of isolation, loneliness, and distress that parents felt was best supported by regular opportunities to connect with class teachers and peers.

"They tried to keep some kind of community, and I think that's what the children are missing."
Toby's mum, Year 3

"It's not just about the work but the connection. Just reminding people that they care about you. That's important."
Harry's mum, Year 5

"I wish my Mum and Dad were keyworkers so I could go back."
Mabel, Year 3

This project was funded by the Higher Education Innovation Fund (HEIF).
To find out more about the Family Adjustment during Covid Epidemic (FACE) Study visit: **www.facestudy.co.uk**, or contact Alison Lacey (**A.Lacey@sussex.ac.uk**) or Dr Kathryn Lester (**K.Lester@sussex.ac.uk**)

FACEStudy
Family Adjustment during Covid Epidemic
